# Artificial intelligence analysis of facial movements and satisfaction in online medical lectures: a cross-sectional study

**DOI:** 10.1186/s12909-026-09175-x

**Published:** 2026-04-15

**Authors:** Mari Miya, Yu Akaishi, Tatsuhiko Anzai, Kenji Suzuki, Masanaga Yamawaki

**Affiliations:** 1 Department of Medical Education Research and Development, Graduate School of Medicine & Dentistry, Institute of Science Tokyo, 1-5-45 Yushima, Bunkyo-ku, Tokyo, 113-8519 Japan; 2 Department of Biostatistics, M＆D Data Science Center, Institute of Science Tokyo, Tokyo, Japan; 3 Institute of Systems and Information Engineering, University of Tsukuba, Ibaraki, Japan

**Keywords:** Facial expression, Blink, Medical student, Artificial intelligence, Online lecture

## Abstract

**Background:**

Detecting learners’ real-time reactions during learning is important. One potential approach is to utilize learners’ nonverbal information. Among nonverbal cues, facial expressions have been shown to influence educational outcomes. Recent technological advances have made quantitative facial expression analysis feasible. In this study, a machine learning tool, a core component of artificial intelligence, was used to quantify subtle facial movements of medical students and examine their relationship with lecture satisfaction.

**Methods:**

Fifth-year medical students attended synchronous and asynchronous online lectures while being recorded. After the sessions, they completed a lecture satisfaction questionnaire. Learners’ facial expressions were analyzed using OpenFace 2.0, a machine learning tool capable of detecting “action units (AUs).” AUs, proposed by Ekman et al., represent subtle facial movements (e.g., AU1 “Inner brow raiser”). In this study, we focused on seven AUs, calculated the total minutes each AU was detected, and analyzed their association with the satisfaction levels reported in the questionnaire.

**Results:**

Regression analysis revealed that overall satisfaction was significantly higher when AU45 (blink) frequency increased during synchronous lectures. A similar analysis using delivery satisfaction as the dependent variable showed that higher AU45 blink rates were significantly associated with greater satisfaction with lecture delivery in synchronous lectures.

**Conclusion:**

Eye blinks, which were more frequent in synchronous lectures, were the only facial cue significantly associated with satisfaction, whereas other facial expressions showed no significant relationship. Further research on learners’ blinking is warranted to better understand real-time responses and to improve the quality of online lectures.

## Background

Online lectures had become widespread after the Coronavirus Disease 2019 pandemic and are now an established learning method [1, 2]. With the development of virtual reality and artificial intelligence (AI) [3, 4], learning styles have become more diverse. While post-class surveys and exams have traditionally been used to assess learning effectiveness, it is also important to detect learners’ real-time reactions during learning. Nonverbal communication (NVC) and other nonverbal cues can provide such insights [5], and we have investigated their potential as indicators of learning effectiveness.

NVC encompasses all behaviors beyond verbal content, including facial expressions, body movements, and vocal tone effectively, every communicative signal except words [6]. In recent years, NVC and nonverbal information have gained prominence in educational research and practice, informing domains such as NVC-based learning support, the influence of teachers’ nonverbal behaviors on learning outcomes, the evaluation of learners’ NVC in medical interviews, and the estimation of learners’ cognitive states (e.g., levels of involvement) through nonverbal indicators [7–9]. Prior research on NVC-informed learning support demonstrates that learners’ facial expressions can signal their comprehension of lectures, thereby enabling instructors to refine instructional delivery [10]. Conversely, learners actively respond to instructors’ facial expressions, posture, gestures, touch, and eye contact, which collectively enrich the learning experience [11, 12]. A systematic review further identified that teachers’ nonverbal immediacy, behaviors that reduce physical and/or psychological distance, correlates positively with heightened student motivation [8].

Within medical education, students’ awareness of NVC improves when they receive structured feedback on interviews conducted with standardized patients [13]. Moreover, existing studies indicate that NVC and nonverbal cues, including eye gaze, facial expressions, and head position, can function as indicators of learner engagement. A review of eye-tracking research emphasized that, although interpretations of cognitive processes in learning contexts require caution, patterns of visual attention derived from eye-tracking metrics offer valuable insight into factors that facilitate or hinder learning performance [14]. Engagement itself denotes learners’ involvement in learning activities and comprises behavioral, emotional, and cognitive dimensions [15]. Empirical work examining engagement through facial expression analysis has identified emotional shifts during lectures by estimating learners’ affective states using automated tools [16]. Associations between student engagement and facial movement, head position, and eye gaze have also been systematically explored [17]. Hu and Gao (2025) further reported that facial expressions of happiness were significantly associated with emotional engagement in online second-language lectures [18].

These three studies employed AI-based analytical tools to process nonverbal data, particularly facial expressions [16–18]. Such techniques enable robust quantitative analysis, with performance surpassing that of earlier manual coding approaches [19, 20]. Collectively, the literature underscores the applicability of NVC for both educational support and assessment, while also highlighting the transformative potential of AI-driven quantitative methods in advancing this line of inquiry. Nevertheless, prior studies have largely focused on global facial expressions or relatively coarse-grained units of analysis. Given that accurate assessment of overall facial expressions typically requires specialized equipment, whereas the identification of discrete facial actions is comparatively accessible, it is pedagogically valuable to examine whether specific facial actions reliably reflect learners’ authentic emotional states and levels of engagement.

Most research on facial expressions has used Ekman et al.’s Facial Action Coding System [21]. It codes fine facial movements, each defined as an “action unit” (AU; e.g., AU1 “inner brow raiser” and AU2 “outer brow raiser”), corresponding to specific facial muscles and muscle groups [22]. AUs have been identified for six basic emotions (anger, disgust, fear, happiness, sadness, and surprise); for example, AU6 (“cheek raiser”) and AU12 (“lip corner puller”) are associated with happiness [23]. Machine learning (ML)-based computer vision algorithms, a core component of AI approaches to facial expression analysis, enable tools such as the open-source OpenFace 2.0 [24]. ML algorithms explore a large space of possible models trained on authentic data to identify the one that optimizes performance [25]. This technology allows objective quantification of learners’ facial movements without burdening participants with wearable devices and may provide a reliable method for assessing cognitive state.

Medical curricula have become increasingly overcrowded in recent years, and the resulting overload has negatively impacted medical students’ learning [26]. Additionally, curricula often require students to learn highly specialized knowledge that is relevant only in certain fields [27]. Another report indicated that medical students were not satisfied with formal study resources selected by the faculty [28]. Given this background, it is crucial for educators to support students in progressing steadily. To address these issues, we posed the following research question: Can learners’ concentration and satisfaction with learning content be assessed in real time and easily using NVC and nonverbal information? To date, although relationships between engagement and NVC have been reported, most studies have evaluated overall facial expressions using facial analysis software. Assessing specific facial movements may be more suitable for real-time, practical evaluation; however, to the best of our knowledge, few studies have examined this approach. Furthermore, identifying specific facial action units that convey learners’ meaningful emotional signals enhances current engagement research utilizing multimodal data, including facial, gaze, and vocal inputs. It enables the verification of relationships between meaningful specific facial movements and multimodal datasets through integrated analytical approaches. Therefore, this study takes an exploratory, hypothesis-generating approach and examines the relationship between medical students’ satisfaction levels and the quantified subtlety of their facial movements using the ML tool OpenFace 2.0 in online classes.

## Methods

### Setting

This cross-sectional study was conducted with 5th-year medical students of the School of Medicine, Tokyo Medical and Dental University (now Institute of Science Tokyo) from August 2022 to August 2023. Online lectures were provided to students participating in clinical training in the Department of General Medicine. Online lectures can be delivered synchronously, where information is sent and received in real time, or asynchronously, where learners access materials at their own pace [29, 30]. In this study, an asynchronous lecture was administered at a designated time.

All lectures were delivered by one of the investigators (Y.A.), and the video materials for the asynchronous lectures were recorded with the same content as the synchronous lectures. Lectures were conducted using a web meeting tool with a maximum of four students at a time. Students were prohibited from taking notes and were asked to refrain from actions that might cover their faces with their hands during the lecture. Before the lecture began, each student was asked to position their face so that it was centered on the screen, and the screen was recorded from the start to the end of the lecture. All participants consented to being filmed during the lecture and completed a questionnaire afterward.

Consent to participate in the study was obtained through an opt-out procedure. Data were excluded if consent was not obtained, if facial expression data could not be collected for more than 10 min due to issues such as falling asleep, or if the principal investigator determined that the image quality was poor or otherwise unsuitable for analysis. Falling asleep was defined as having the eyes closed with the head tilted downward for an extended period or dozing off. The study protocol, including post-hoc consent, was approved by the ethical review board of Tokyo Medical and Dental University (Approval No.: C2022-050).

### Participants

**Fig. 1:**
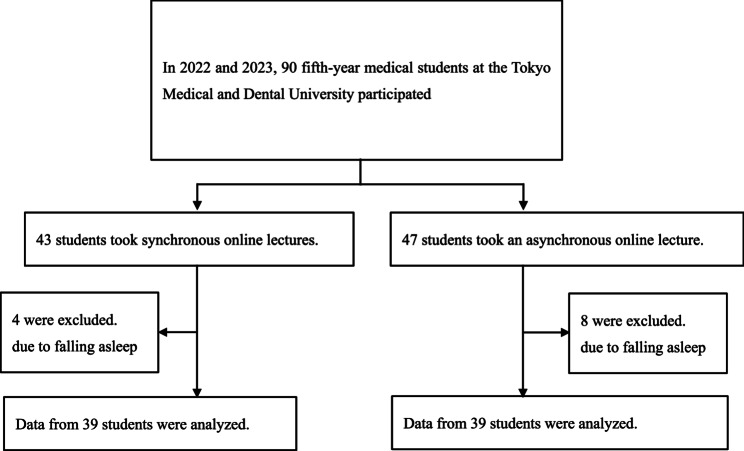
Study flow diagram

Participants were 5th-year medical students attending clinical training in the mornings, followed by lectures in the afternoons. Ninety students attended online lectures: 43 in the synchronous group (live lectures) and 47 in the asynchronous group (recorded lectures). Students were alternately assigned to the synchronous and asynchronous groups, and after the second round, adjustments were made to ensure that the difference in group sizes did not exceed four students. Data from four students in the synchronous group and eight students in the asynchronous group were excluded due to insufficient data, such as falling asleep within the first 10 min. Thus, data from 39 students in each group were analyzed (Fig. 1).

### Lectures

The lecture content on sore throats covered bacterial infections, including group A Streptococcus; viral infections, such as respiratory viruses and Epstein–Barr virus; and diseases requiring urgent attention. For the 30-minute lectures, the first 15 min were delivered as either synchronous or asynchronous. To ensure equal learning opportunities, students who attended synchronous lectures first (synchronous group) received asynchronous lectures in the second half, and vice versa. Only the first 10 min of data were used in the analysis to align the timing across groups.

### Lecture satisfaction questionnaire

A lecture satisfaction questionnaire was developed based on the lecture evaluation form by Hytham et al. [31]. Ten questions were included, with responses recorded on a 5-point scale (5 = “Yes,” 1 = “No”; Table 1). The average score of all items was calculated to determine the overall satisfaction. Questions 1–7 focused on lecture content, while Questions 8–10 addressed delivery method. Mean scores were calculated for each section. As a subcategory of overall satisfaction, the mean score for content-related questions was defined as content satisfaction, and the mean score for delivery-related questions was defined as delivery satisfaction. The Cronbach’s alpha coefficient was 0.88 for the full questionnaire, 0.83 for content-related items, and 0.82 for delivery-related items.

**Table 1:** Lecture satisfaction questionnaire*

Content	
Question 1	The objectives were clear and were achieved.
Question 2	This lecture was appropriate for my level of training.
Question 3	This lecture presented new information.
Question 4	The key points were easy to understand.
Question 5	This lecture will help me in patient care.
Question 6	The quantity of information covered was appropriate.
Question 7	This lecture stimulated my interest in the subject matter.
Delivery	
Question 8	The slides improved the presentation.
Question 9	The lecture was well organized.
Question 10	The presentation was dynamic and interesting.

* Questions 1–7 pertain to lecture content, while questions 8–10 address the method of delivery. The questionnaire was developed based on the lecture evaluation form created by Hytham et al. [31]

### OpenFace 2.0

The edited videos were analyzed using OpenFace 2.0, an ML-based facial expression analysis tool that can automatically detect AUs. OpenFace 2.0 has demonstrated an average concordance correlation coefficient of 0.73 relative to human-coded ratings in DISFA, a dataset comprising spontaneous facial expressions [24]. In a study of Japanese individuals, AU detection accuracy using OpenFace 2.0 was generally high, though it varied across AUs, based on a relatively small dataset [32]. The video used in this study had a frame rate of 30 frames per second, and for each frame, the tool outputs whether each AU is detected. For each AU, the total number of frames detected was divided by 1,800 frames per minute to calculate the total minutes for each detected AU.

In this study, a satisfied expression was considered similar to a happy face, and AU6 (cheek raiser) and AU12 (lip corner puller), which are associated with happiness, were analyzed. A previous study reported that facial expressions resembling anger often indicate deep focus [33]. A serious, concentrated expression was therefore considered similar to an angry face, and AU4 (brow lowerer), AU5 (upper lid raiser), AU7 (lid tightener), and AU23 (lip tightener) were assessed. Additionally, AU45 (blink), an indicator of blinking, was analyzed.

### Statistical analysis

Means and standard deviations were calculated for each item of the lecture satisfaction questionnaire, overall satisfaction scores, their subscales (content satisfaction and delivery satisfaction), and the seven selected AUs. For both synchronous and asynchronous groups, simple linear regression analyses were conducted with overall satisfaction as the dependent variable and each of the seven AUs as independent variables. Multiple regression analyses were also performed, adjusting for gender and other AUs. Similar single and multiple regression analyses were conducted for the subscales. Standard assumptions for regression analyses were assessed. Normality of residuals was evaluated via visual inspection of the Q–Q plots and was deemed satisfactory. Homoscedasticity was confirmed through residual plots. Statistical analyses were performed using Stata version 17.0 (Stata Corp LLC, College Station, TX, USA), with a *p*-value of 0.05 set as the threshold for statistical significance.

## Results

### Study characteristics

Table 2 presents the background information of the study participants. The distributions of gender and academic year, as well as the mean values for each action unit (AU4, 5, 6, 7, 12, 23, and 45), were similar between the synchronous and asynchronous groups. Table 3 shows that overall satisfaction and delivery satisfaction were significantly higher in the synchronous group compared to the asynchronous group (overall satisfaction: 4.65 vs. 4.42; delivery satisfaction: 4.50 vs. 4.06).

**Table 2:** Characteristics of the 5th year medical students and action units*

Characteristic	Type of Online Lectures	
	Synchronous (*n* = 39)	Asynchronous (*n* = 39)
Male gender, *n* (%)	26 (66.7)	26 (66.7)
Academic year, *n* (%)		
2022	20 (51.3)	20 (51.3)
2023	19 (48.7)	19 (48.7)
AU [min]**, mean (SD)		
AU4	5.81 (4.00)	5.35 (4.33)
AU5	4.34 (3.65)	4.10 (3.72)
AU6	0.14 (0.39)	0.25 (0.96)
AU7	4.98 (4.07)	4.83 (4.35)
AU12	0.21 (0.62)	0.25 (0.74)
AU23	2.74 (3.10)	3.40 (3.68)
AU45	2.27 (0.55)	2.43 (0.70)

* SD denotes standard deviation.

** AU denotes action unit. The data represent the number of minutes detected by OpenFace2.0 during the first 10 min of the lecture. Ekman et al. (1978) defined facial movements as AUs (Ekman et al., 1978). AU4 is “brow lowerer,” AU5 is “upper lid raiser,” AU6 is “cheek raiser,” AU7 is “lid tightener,” AU12 is “lip corner puller,” AU23 is “lip tightener,” and AU45 is “blink.”

**Table 3:** Characteristics of lecture satisfaction*

**Characteristic**	**Type of Online Lectures**	
	Synchronous (*n* = 39)	Asynchronous (*n* = 39) *P*-value
Question**, mean (SD)		
Question 1	4.85 (0.43)	4.69 (0.57) 0.18
Question 2	4.54 (0.72)	4.59 (0.64) 0.74
Question 3	4.79 (0.41)	4.62 (0.75) 0.19
Question 4	4.82 (0.45)	4.62 (0.75) 0.15
Question 5	4.85 (0.37)	4.77 (0.48) 0.43
Question 6	4.64 (0.63)	4.54 (0.68) 0.49
Question 7	4.56 (0.60)	4.18 (0.72) 0.012***
Question 8	4.72 (0.60)	4.44 (0.85) 0.096
Question 9	4.77 (0.54)	4.59 (0.68) 0.20
Question 10	4.00 (0.86)	3.18 (1.05) < 0.001***
Overall satisfaction, mean (SD)	4.65 (0.40)	4.42 (0.51) 0.028
Content satisfaction, mean (SD)	4.73 (0.37)	4.58 (0.47) 0.11
Delivery satisfaction, mean (SD)	4.50 (0.57)	4.06 (0.75) 0.005***

* SD denotes standard deviation

** A 5-point Likert scale was applied to the questionnaire, with 5 indicating “agree” and 1 indicating “disagree.” Overall satisfaction represents the mean score of all 10 items in the questionnaire. Content satisfaction represents the mean score of Questions 1–7, and delivery satisfaction represents the mean score of Questions 8–10

*** *P* < 0.05

### Association between overall satisfaction and AUs

In the synchronous group, overall satisfaction was significantly higher as the duration of AU45 (blink) increased, both in the simple and multiple regression analyses (simple regression coefficient 0.33, 95% confidence interval [CI]: 0.12–0.55, *p* = 0.003; multiple regression coefficient 0.25, 95% CI: 0.022–0.48, *p* = 0.033; Table 4). No significant associations were observed between overall satisfaction and other AUs in the synchronous group or with any AUs in the asynchronous group.

**Table 4:** Association between overall satisfaction and action units in synchronous and asynchronous groups*

	Simple regression			Multiple regression**		
	Coefficient	95% CI	*P*-value	Coefficient	95% CI	*P*-value
Synchronous group (*n* = 39)						
AU4 [min]	−0.015	[− 0.048, 0.018]	0.366	−0.006	[− 0.042, 0.030]	0.739
AU5 [min]	0.002	[− 0.034, 0.039]	0.898	0.004	[− 0.035, 0.042]	0.847
AU6 [min]	0.068	[− 0.271, 0.407]	0.688	0.023	[− 0.309, 0.355]	0.889
AU7 [min]	0.001	[− 0.032, 0.033]	0.963	−0.010	[− 0.049, 0.029]	0.600
AU12 [min]	−0.195	[− 0.401, 0.010]	0.062	−0.178	[− 0.386, 0.031]	0.092
AU23 [min]	−0.025	[− 0.067, 0.017]	0.234	−0.013	[− 0.055, 0.029]	0.531
AU45 [min]	0.332	[0.119, 0.546]	0.003***	0.251	[0.022, 0.480]	0.033***
Asynchronous group (*n* = 39)						
AU4 [min]	0.012	[− 0.027, 0.051]	0.546	0.014	[− 0.043, 0.070]	0.628
AU5 [min]	−0.002	[− 0.048, 0.044]	0.941	0.005	[− 0.050, 0.060]	0.861
AU6 [min]	0.030	[− 0.148, 0.207]	0.735	−0.004	[− 0.214, 0.205]	0.967
AU7 [min]	0.015	[− 0.024, 0.054]	0.431	0.005	[− 0.055, 0.065]	0.871
AU12 [min]	0.044	[− 0.184, 0.273]	0.696	0.073	[− 0.219, 0.364]	0.615
AU23 [min]	−0.025	[− 0.071, 0.020]	0.270	−0.032	[− 0.090, 0.026]	0.267
AU45 [min]	−0.073	[− 0.314, 0.169]	0.546	−0.008	[− 0.322, 0.306]	0.960

* CI denotes the confidence interval. AU4 is “brow lowerer,” AU5 is “upper lid raiser,” AU6 is “cheek raiser,” AU7 is “lid tightener,”AU12 is “lip corner puller,” AU23 is “lip tightener,” and AU45 is “Blink.”

** Adjusted for male gender, other six AUs [min]

*** *p* < 0.05

### Association between satisfaction subscales and AUs

In the synchronous group, content satisfaction was significantly higher with increasing AU45 duration in the simple regression analysis, but this association was not significant in the multiple regression analysis (simple regression coefficient 0.26, 95% CI: 0.050–0.47, *p* = 0.016; multiple regression coefficient 0.18, 95% CI: −0.038 to 0.40, *p* = 0.10; Table 5). Delivery satisfaction was significantly higher with increasing AU45 duration in both the simple and multiple regression analyses (simple regression coefficient 0.51, 95% CI: 0.21–0.81, *p* = 0.002; multiple regression coefficient 0.41, 95% CI: 0.088–0.74, *p* = 0.015; Table 5). No significant associations were found between satisfaction subscales and other AUs in either the synchronous or asynchronous groups.

**Table 5:** Association between subscale and action units in the synchronous group (*n* = 39)*

	Simple regression			Multiple regression**		
	Coefficient	95% CI	*P*-value	Coefficient	95% CI	*P*-value
Content satisfaction						
AU4 [min]	−0.170	[− 0.048, 0.014]	0.266	−0.011	[− 0.046, 0.023]	0.517
AU5 [min]	0.013	[− 0.020, 0.047]	0.430	0.014	[− 0.023, 0.051]	0.459
AU6 [min]	0.087	[− 0.229, 0.403]	0.581	0.032	[− 0.288, 0.352]	0.839
AU7 [min]	0.002	[− 0.028, 0.033]	0.888	−0.003	[− 0.041, 0.035]	0.875
AU12 [min]	−0.166	[− 0.360, 0.028]	0.091	−0.168	[− 0.369, 0.033]	0.098
AU23 [min]	−0.012	[− 0.051, 0.028]	0.559	−0.002	[− 0.042, 0.039]	0.927
AU45 [min]	0.258	[0.050, 0.465]	0.016***	0.183	[− 0.038, 0.403]	0.100
Delivery satisfaction						
AU4 [min]	−0.009	[− 0.057, 0.038]	0.689	0.006	[− 0.045, 0.058]	0.797
AU5 [min]	−0.019	[− 0.071, 0.033]	0.460	−0.016	[− 0.070, 0.039]	0.559
AU6 [min]	0.061	[− 0.427, 0.550]	0.800	0.030	[− 0.441, 0.501]	0.898
AU7 [min]	−0.0005	[− 0.047, 0.046]	0.983	−0.026	[− 0.081, 0.030]	0.353
AU12 [min]	−0.260	[− 0.557, 0.038]	0.085	−0.197	[− 0.493, 0.100]	0.185
AU23 [min]	−0.053	[− 0.112, 0.006]	0.076	−0.035	[− 0.095, 0.025]	0.238
AU45 [min]	0.508	[0.207, 0.809]	0.002***	0.413	[0.088, 0.738]	0.015***

* CI denotes the confidence interval. AU4 is “brow lowerer,” AU5 is “upper lid raiser,” AU6 is “cheek raiser,” AU7 is “lid tightener,” AU12 is “lip corner puller,” AU23 is “lip tightener,” and AU45 is “blink.”

** Adjusted for male gender, other six AUs [min]

*** *P* < 0.05

## Discussion

In this exploratory study, the main finding was that learner satisfaction was associated with a higher eye-blink rate during synchronous online lectures. Another key finding was that no significant relationship was observed between other facial movements and satisfaction. Regarding the subscales, delivery satisfaction increased as the number of AU45 detections increased in the synchronous group. These results suggest that learner satisfaction may be more closely linked to eye blinks than to facial expressions, highlighting the potential of blinks as a nonverbal marker of learning engagement.

The main finding of this study was that participants with higher blink rates reported significantly greater lecture satisfaction. To date, although the association between changes in blink rate during short scenes and concentration or interest has been examined [34, 35], little is known about how blinking relates to engagement over a 10-minute session. Clarifying this relationship is therefore one of the key contributions of this study. The finding that eye-blink information reflected learners’ feelings more strongly than facial expressions aligns with previous research showing that, compared with using facial expressions alone, incorporating eye gaze provided better insight into learners’ engagement in collaborative learning environments [36]. Given the fundamental differences between previous studies and ours, direct comparisons are not straightforward; however, both studies highlight the important role of eye-related information in educational settings.

Blinking can be categorized into three types: voluntary, reflexive, and spontaneous. Spontaneous blinking occurs involuntarily without direct external stimuli and helps maintain lubrication of the ocular surface [37, 38]. The blinking observed in this study was spontaneous, occurring unconsciously during the lectures. Several factors influence spontaneous blinking. A review reported that sex differences in blink rate range from none to higher rates in females than in males. No clear conclusions have been drawn regarding age, although blink rate appears to be stable into adulthood [39]. In this study, results were equivalent after adjustment for sex and age, suggesting that potential confounding effects were partially controlled. Interestingly, eye-blink rate has also been considered a noninvasive, indirect marker of central dopamine (DA) function [39–41]. As DA is related to working memory, reinforcement learning, and cognitive control [42–44], blinking may reflect cognitive function during learning, mediated by DA. However, some studies have questioned this relationship [45, 46], and interpretations should be made with caution.

To date, although the mutual process in the relationship between central function and blinking has not been fully elucidated, blink rate is influenced by situational and temporal factors and can be categorized into contexts of suppression or increase related to engagement. Overall, blink rate decreases during reading [47]. Similarly, attention-demanding scenes suppress blink rate during the viewing of films and videos [34, 48]. Shin et al. reported that participants identified a memorable scene after watching a film, and the blink rate was suppressed during that scene. Performance on an episodic memory test administered four weeks later was significantly associated with the timing of blink suppression [35]. On the other hand, scenes in which comprehension is disrupted while reading and watching videos increase blink rate [49, 50]. In cognitive tasks without visual attention, scenes with higher cognitive load were associated with increased blink rate [51]. Furthermore, eye blinks increased when information was maintained or updated during a working memory task [52, 53]. Working memory tasks refer to the temporary storage and processing of information. In summary, prior research demonstrates that blink rate is suppressed during periods of visual concentration, whereas it increases during processes involving the organization and integration of information.

The lecture segment analyzed in this study covered clinical content, including the Centor criteria for diagnosing group A Streptococcal pharyngitis and key complications to watch for after diagnosis. Post-lecture reflection indicated that the Centor criteria were new to the participants. Another segment addressed how to organize and integrate previously acquired knowledge for application in a real clinical setting. In our study, we estimated that blink suppression during attention-demanding scenes was limited, whereas blink rate increased in scenes involving the organization and integration of knowledge. Hence, participants who judged the lecture to be valuable and engaged more in knowledge integration reported higher lecture satisfaction, which was associated with higher blink rate. However, these interpretations involve a degree of speculation. Future research is required to directly examine changes in blink rate as learners encounter new knowledge, as well as during the integration and organization of foundational medical knowledge into clinical practice.

Additionally, the subitem of lecture satisfaction related to delivery was significantly associated with eye blinking in synchronous lectures. Previous research reported that lecturers can adjust their NVC by estimating learners’ understanding from their facial expressions during online lectures [10]. Moreover, increased blink rates have been reported during speech and while listening to a paragraph for memorization [54]. Settings in online synchronous lectures resemble these conditions, and lecturers adjusted their delivery to make content easier to understand and memorize, which may have contributed to an increased blink rate with increasing satisfaction. In this study, it is important to note that the relationship between delivery satisfaction and blink rate likely exerts a greater influence on overall satisfaction than content satisfaction, which demonstrated no significant effect in the multiple regression analysis. Therefore, the finding that learner satisfaction was associated with a higher eye-blink rate may not be attributable to changes in blink rate during the integration and organization of knowledge, but rather to the social interactions between the lecturer and the learner.

Facial movements were not related to learner satisfaction in this study. Several previous studies have examined the association of learners’ facial expressions with engagement or emotions during lectures [16, 18]. In online lectures, real-time analysis of learners’ facial expressions through webcams can evaluate their level of concentration and engagement [10, 17]. However, these studies analyzed overall facial expressions, whereas our study examined changes in specific facial movements. It is important to note that the analyzed video data were limited to the first 10 min of each lecture, and participants were informed in advance that they were being recorded. These factors may have influenced the participants’ natural facial expressions. Awareness of being observed can lead individuals to adopt a more objective or self-conscious perspective toward their behavior. Heightened self-awareness increases sensitivity to discrepancies between actions and perceived social norms, which can suppress spontaneous facial expressions and emotional displays [55]. Consequently, the awareness of being recorded may have reduced expressiveness and limited the accurate representation of learners’ satisfaction.

AI has improved significantly in recent years, and many studies on AI-supported medical education have been published. Studies have reported the effectiveness of AI feedback as an alternative to attending physicians for clinical reasoning training among medical students [56], as well as the educational effectiveness of medical interview training with AI-simulated patients [57]. The effectiveness of AI-based automatic assessment for the Objective Structured Clinical Examination has also been reported [58, 59]. Reports on AI-supported learning indicate that teaching and evaluation can be conducted to a certain standard even when human resources are limited. Prior educational research outside medical education has demonstrated the effectiveness of AI-based multimodal learning analytics incorporating facial, gaze, and voice data, among others. A review article highlighted that using multimodal learner data provides advantages such as deeper insights into the learning process, personalized learning, and real-time feedback [60]. Based on the blink characteristics examined in this study, similar educational benefits are anticipated. Educators could enhance learning opportunities by monitoring changes in learners’ blink rates in real time.

This study has several limitations. First, interpretations regarding learners’ attentional fluctuations (e.g., focus and concentration during the lecture) and variations in cognitive load (e.g., the integration and organization of knowledge) involve a degree of speculation, for which further investigation is warranted. Accordingly, this study is best characterized as hypothesis-generating rather than confirmatory, given its highly novel and exploratory nature. Second, the study focused on a medical lecture at a single institution in Japan, limiting generalizability. Multicenter studies and lectures in other disciplines are needed to validate and extend these findings. Third, although an association was observed between eye blinks and lecture satisfaction, this specifically relates to AU45 blinks detected by the ML tool OpenFace 2.0. While OpenFace 2.0 is more accurate than random AU detection, it is not 100% precise [40]; nevertheless, it was considered appropriate for the study objectives. Fourth, the study analyzed blink detection data as a whole and did not assess increases or decreases in blink frequency throughout the lecture, which warrants further investigation. Future research could explore how prompting and questioning strategies influence concentration, using NVC as an indicator. Finally, factors such as dry eye and eye strain may influence blink rate [41]. Individuals using computers for extended periods often experience dry eye [42]. Additionally, with regard to physiological factors associated with screen use, eye strain resulting from visual display terminal work is known to increase blink rate [61]. In our study, participants attended the lecture using computers. Therefore, the observed increase in blink frequency may have been partly caused by dry eyes and eye strain associated with computer use. However, these influences were likely limited because the lecture was only 10 min long, making a substantial change in blink rate unlikely, though it cannot be completely ruled out. Future studies on blink behavior should consider assessing dry eye.

A significant relationship was observed between learner satisfaction and blinking during synchronous lectures, whereas no significant correlation was found with other facial expressions, suggesting that blinking may reflect learners’ responses during the lecture. Delivery satisfaction ratings were higher when blinking increased. These findings indicate that lecturers could potentially gauge learner engagement and responses in real time during synchronous classes—both in-person and online—by monitoring blinking cues.

## Data Availability

The data cannot be shared openly but are available on request. Although personally identifiable information is not available due to privacy or ethical restrictions.

## Abbreviations

AI: Artificial intelligence
AU: Action unit
CI: Confidence interval
DA: Dopamine
ML: Machine learning
NCV: Nonverbal communication
